# Supplementary material for: Evidence for low density holes in Jupiter’s ionosphere
Source: Nat Commun. 2019 Jun 21;10:2751. doi: 10.1038/s41467-019-10708-w (PMC6588597; doi:10.1038/s41467-019-10708-w)
Supplement: Supplementary file 1 — Supplementary Information [file 41467_2019_10708_MOESM1_ESM.pdf]

Supplementary Information of

Evidence for low density holes in Jupiter's ionosphere

Masafumi Imai[1], Ivana Kolmašová[2,3], William S. Kurth[1], Ondřej Santolík[2,3], George B. Hospodarsky[1], Donald A. Gurnett[1], Shannon T. Brown[4], Scott J. Bolton[5], John E. P. Connerney[6,7] & Steven M. Levin[4]

[1]Department of Physics and Astronomy, University of Iowa, Iowa City, Iowa, USA. [2]Department of Space Physics, Institute of Atmospheric Physics, The Czech Academy of Sciences, Prague, Czechia. [3]Faculty of Mathematics and Physics, Charles University, Prague, Czechia. [4]Jet Propulsion Laboratory, California Institute of Technology, Pasadena, California, USA. [5]Space Science and Engineering Division, Southwest Research Institute, San Antonio, Texas, USA. [6]Space Research Corporation, Annapolis, Maryland, USA. [7]NASA Goddard Space Flight Center, Greenbelt, Maryland, USA.

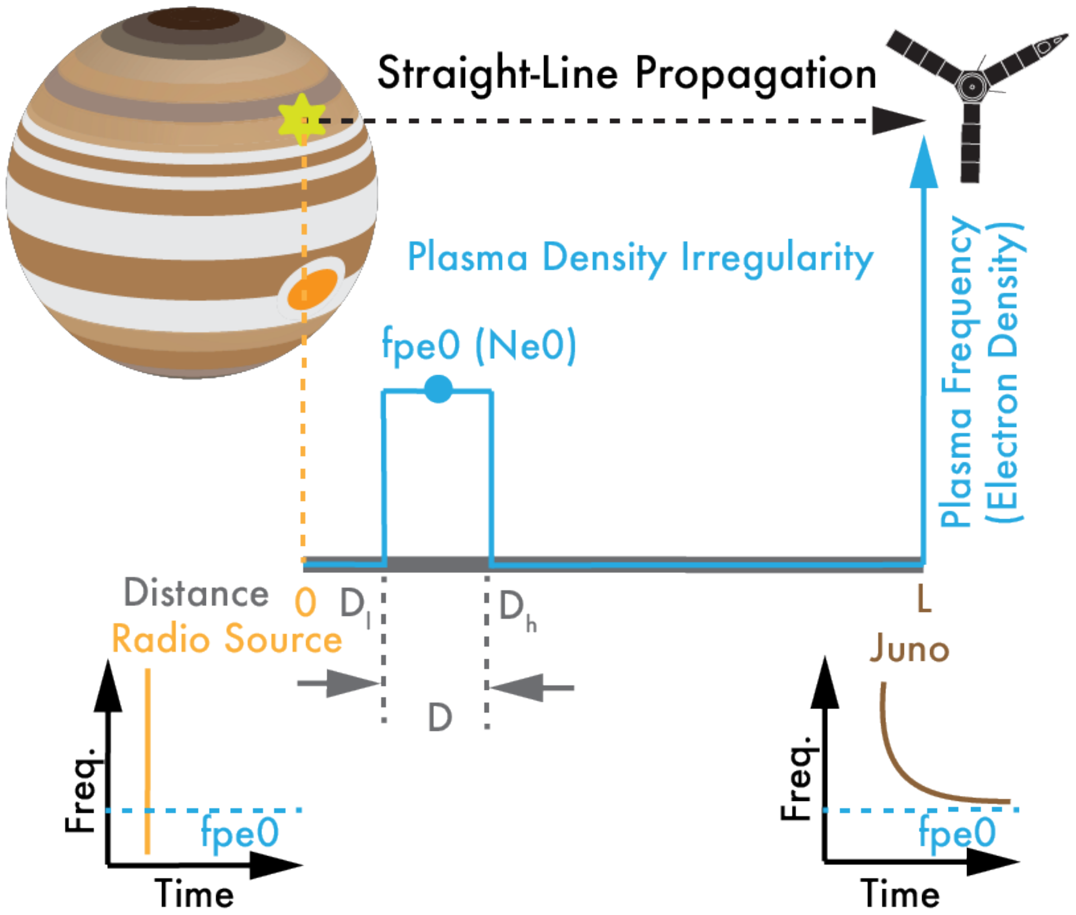

Supplementary Figure 1 | Schematic of the O mode straight-line propagation model. Juno, an unknown radio source, and a plasma density irregularity are collinear. The impulse propagates from the source through the plasma irregularity and continues on to Juno, where the dispersed signal is detected.

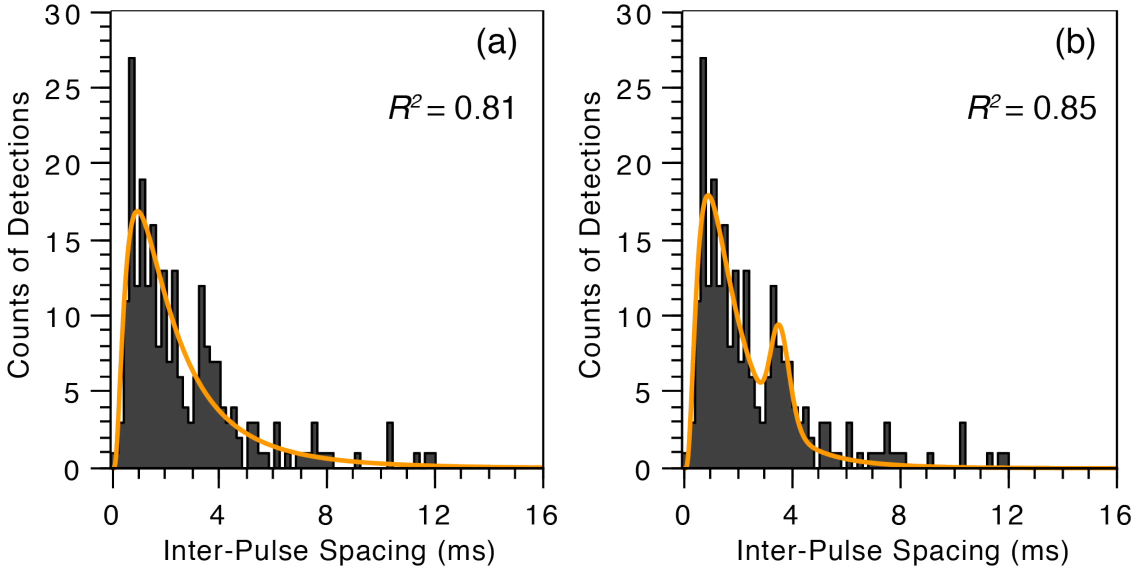

Supplementary Figure 2 | Histograms of inter-pulse spacing. The fittings are made with (a) one modified log-normal distribution and (b) two modified log-normal distributions. The modified log-normal distribution is $f(x) = \sum_{i=0}^{n} \frac{A_i}{\sqrt{2}S_i x} \exp\left(-\frac{(\ln x - M_i)^2}{2{S_i}^2}\right)$. The best-fitting results are $A_0 = 46.96$, $S_0 = 0.85$, and $M_0 = 0.63$ for (a) at $n = 0$, and $A_{0,1} = (38.49, 5.46)$, $S_{0,1} = (0.74, 0.10)$, and $M_{0,1} = (0.42, 1.27)$ for (b) at $n = 1$. The coefficient of determination is defined as $R^2 = 1 - \sum_{i=1}^{80} \frac{(y_i - m_i)^2}{(y_i - \bar{y})^2}$, where y_i and $\bar{y}$ are the observation data in the histogram and their mean value, and m_i is the model data computed from the best-fitted distributions.

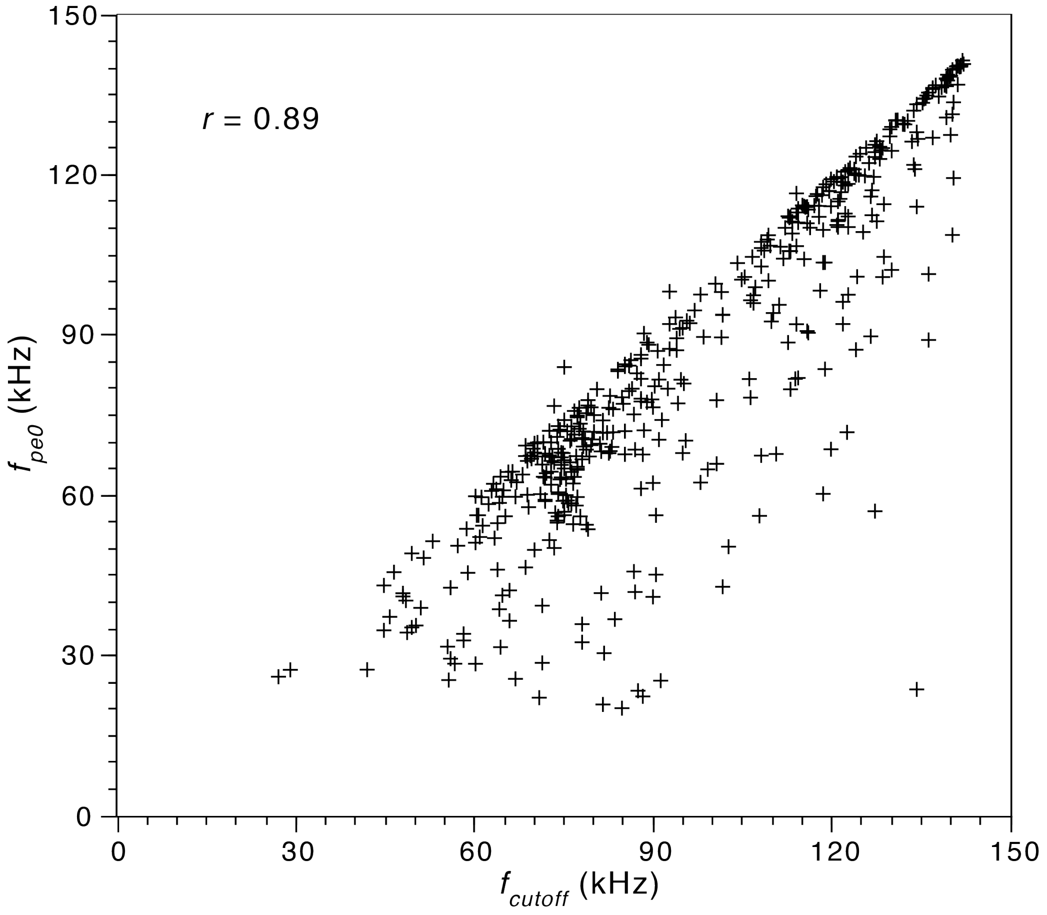

Supplementary Figure 3 | Comparison of f_{pe0} and f_{cutoff}. The former was estimated from the O mode propagation model and the latter was measured from the spectrograms. Pearson correlation coefficient *r* is 0.89, a positive correlation.

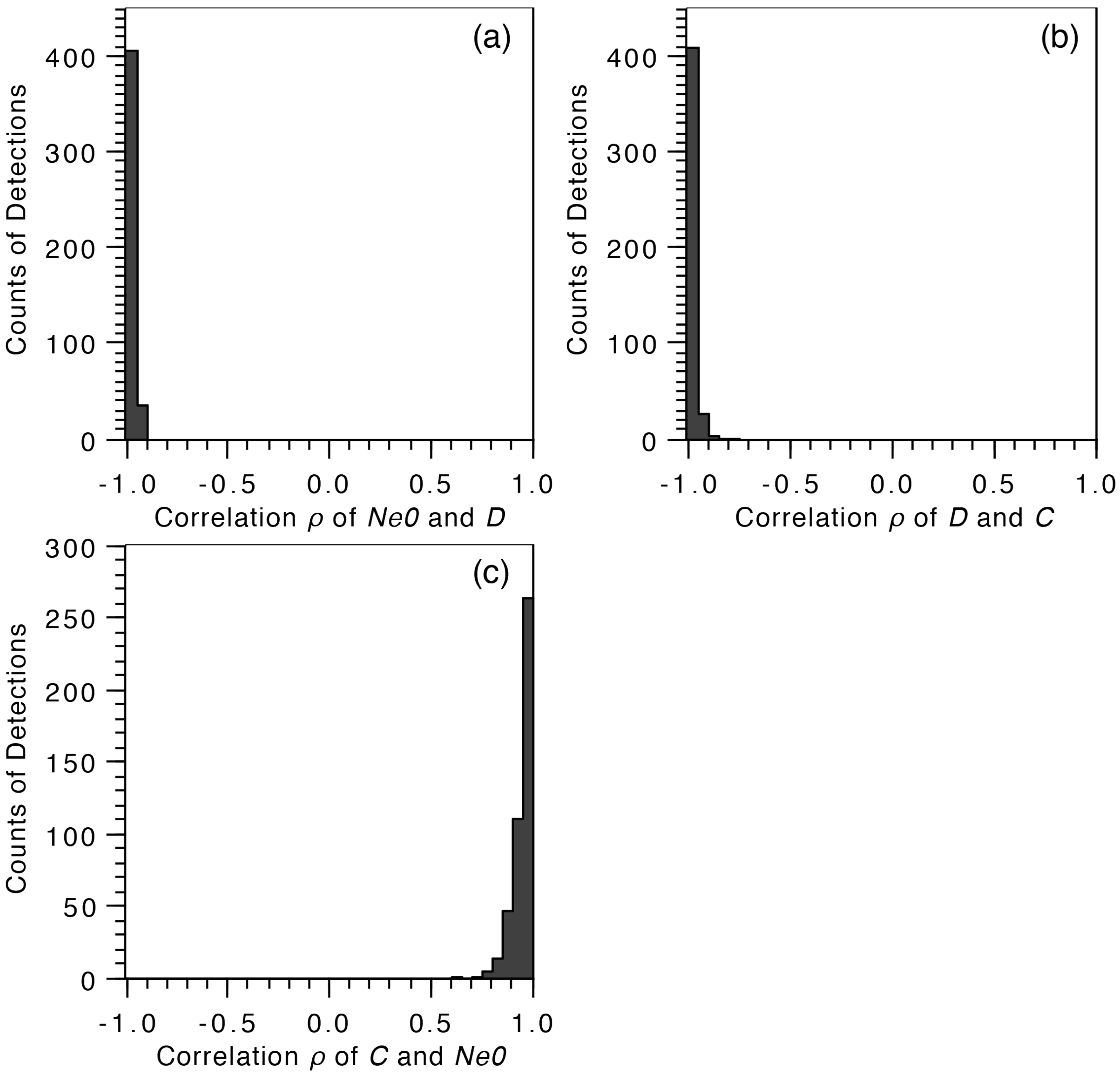

Supplementary Figure 4 | Histograms of correlations of the model parameters. The correlation is defined as $\rho = \frac{\sum_{i=0}^{n}(x_i-\bar{x})(y_i-\bar{y})}{\sqrt{\sum_{i=0}^{n}(x_i-\bar{x})^2}\cdot\sqrt{\sum_{i=0}^{n}(y_i-\bar{y})^2}}$, where x_i and y_i are the model variables of N_{e0} and D for (a), D and C for (b), and C and N_{e0} for (c), respectively. $\bar{x}$ and $\bar{y}$ are the mean values of the corresponding variables.

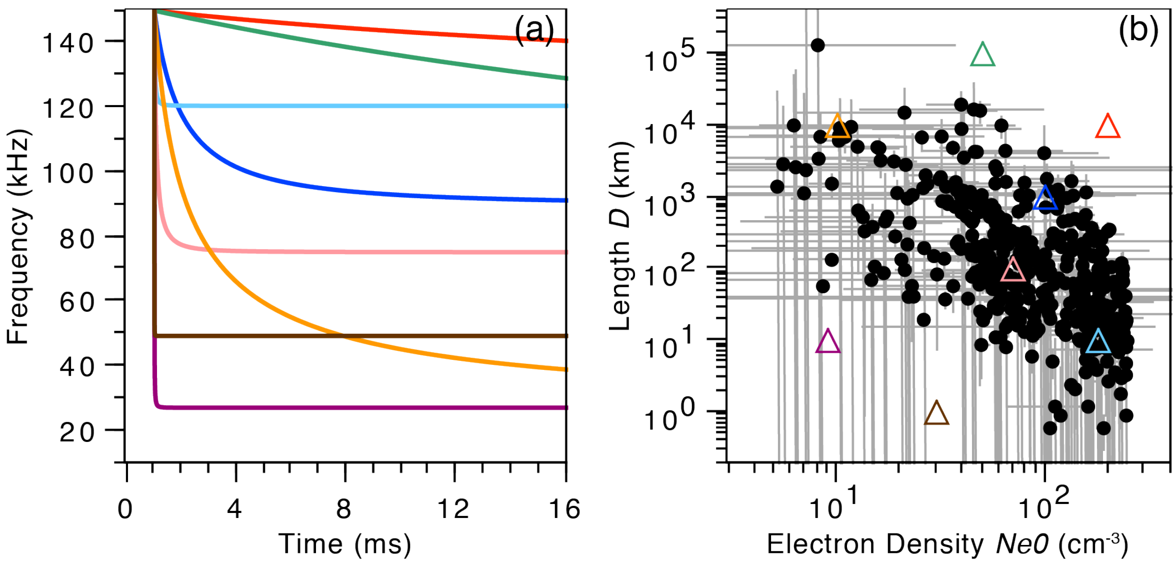

Supplementary Figure 5 | Simulated dispersed pulses using the propagation model. (a) The red, green, blue, sky blue, pink, orange, purple, and brown curves represent the cases of (200, 10^4), (50, 10^5), (100, 10^3), (180, 10), (70, 10^2), (10, 10^4), (9, 10) and (30, 1), respectively, where the listed values refer to (N_{e0} in cm^{-3}, D in km). Note that the initial arrival time of all examples were sorted at 1 ms. (b) The plot is the same as in Figure 2c, but the triangles show the corresponding colour dispersed pulses in (a). The grey error bar indicates one standard deviation (68% confidence interval) of N_{e0} and D. It is clear that the O mode straight-line propagation model allows to express various types of spectral structures but the nature of JDPs appears the limited sets of D and N_{e0} (e.g. the orange, blue, pink, and sky blue dispersed pulses). In contrast, the green, red, purple, and brown dispersed pulses were not detected in our study.

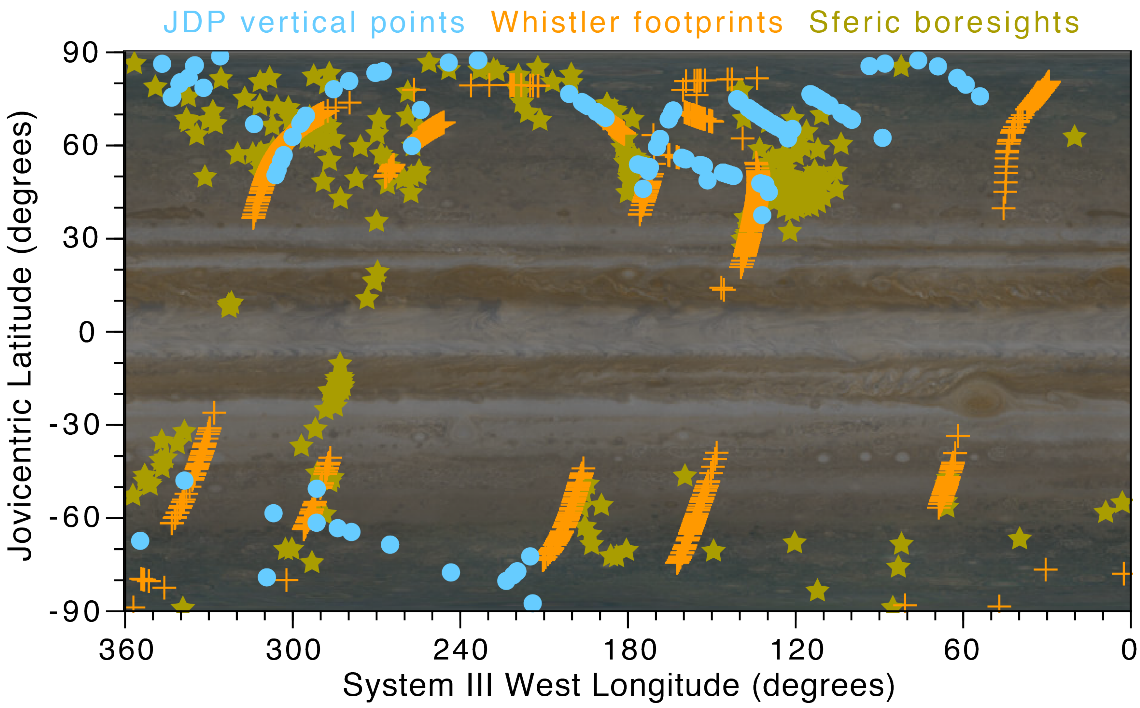

Supplementary Figure 6 | Comparison of JDPs, lightning-induced whistlers and sferics. The format is the same as in Fig. 3 but for the common periods from perijove 1 through 8. We use data during intervals when the Waves LFR-Hi mode observations were available. The Jovian image was provided by NASA/JPL-Caltech/SSI/SwRI/MSSS/ASI/INAF/JIRAM/Björn Jónsson (http://www.planetary.org/multimedia/space-images/jupiter/merged-cassini-and-juno.html).

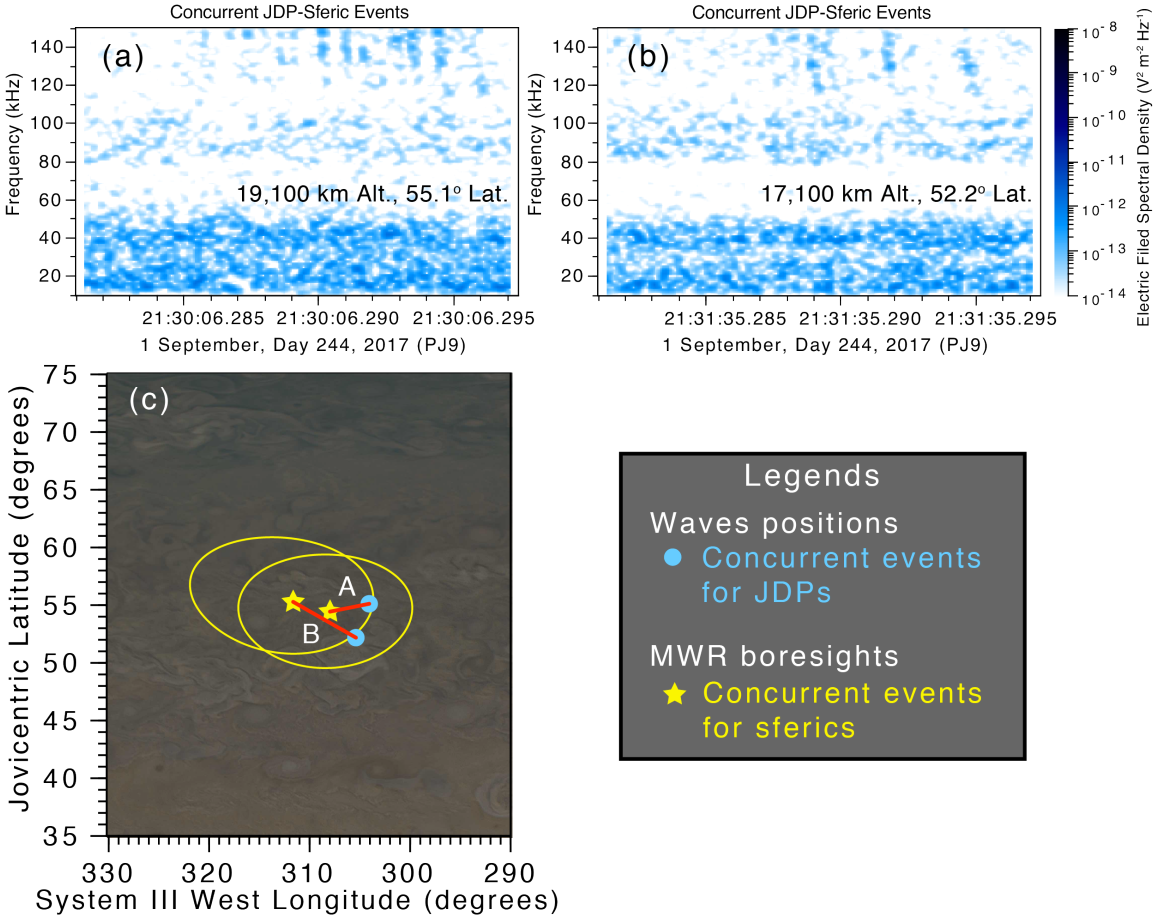

Supplementary Figure 7 | Concurrent JDP-sferic events. The time spans are (a) 21:30:06.281-06.297384 and (b) 21:31:35.281-35.297384 on 1 September, 2017. (c) The locations of JDPs and sferics for these events are depicted. We found 39 MWR sferic 100-ms events that overlap with Waves 16.384-ms snapshots but there were only two Waves snapshots in which we detected JDPs. The yellow ellipses indicate the MWR 17° beam half-angle projected onto Jupiter corresponding to about 90% of the received power[22]. One reason for a small number of concurrent event is a different propagation scenario. While the 600-MHz sferics propagate freely through the dense ionosphere, the JDPs can be seen only when ionospheric holes are present. Another possibility is an unclear number of sferics during a Waves snapshot. While Waves can clearly capture individual JDPs in a 16.384-ms waveform snapshot, there is no way to identify exactly when individual sferics occur within the MWR sferic 100-ms integration time. These observational and instrumental restrictions most probably limit the number of the concurrent JDP and sferic events. The Jovian image in (c) was provided by NASA/JPL-Caltech/SSI/SwRI/MSSS/ASI/INAF/JIRAM/Björn Jónsson (http://www.planetary.org/multimedia/space-images/jupiter/merged-cassini-and-juno.html).

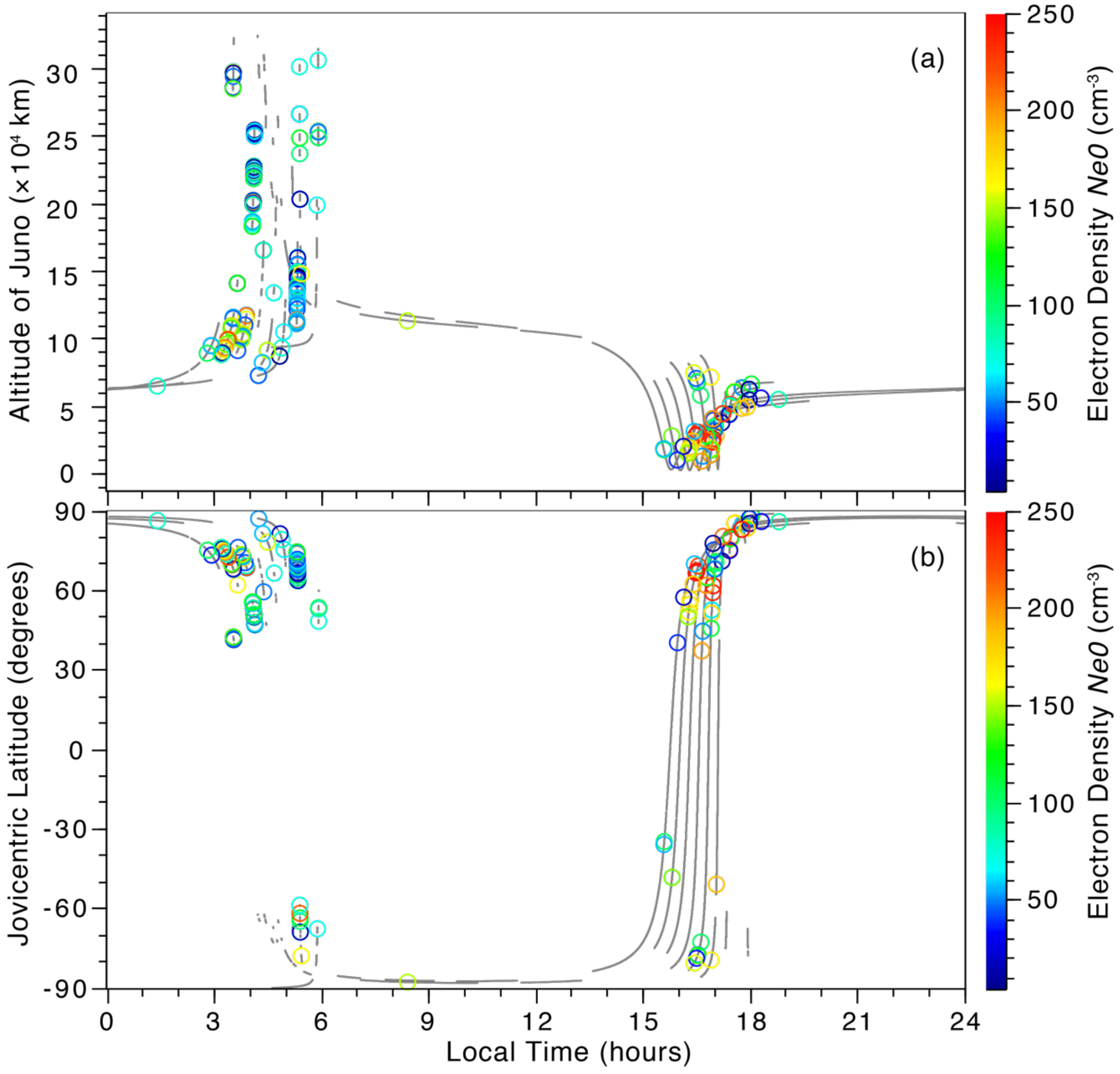

Supplementary Figure 8 | Estimated electron density N_{e0} from JDPs. These distributions in local time are plotted as a function of (a) altitude of Juno and (b) Jovicentric latitude at Juno. The grey lines correspond to the Waves LFR-Hi observational coverage from PJ1 through PJ9. Although almost all JDP detections are captured on Jupiter's dawn and dusk sides, one JDP is detected on the day side near 90° latitude, which means that this JDP can come from a thunderstorm in any local time. Hence, Juno's detections of JDPs are near the terminator, which supports possible radio sources of JDPs and ionospheric holes on Jupiter's night side.

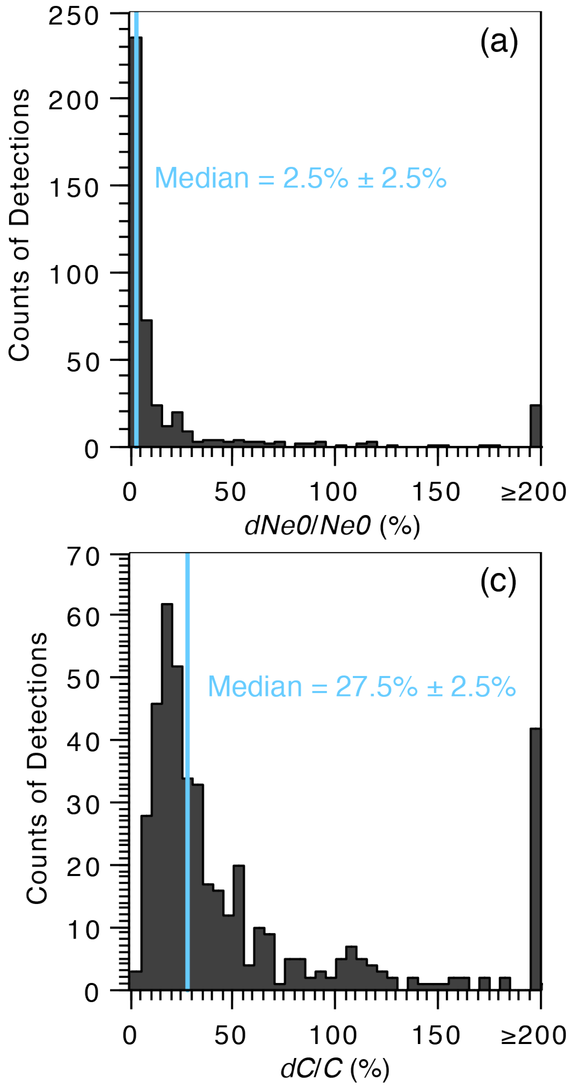

Supplementary Figure 9 | Histograms of fractions of the model parameters. Three parameters dN_{e0}, dD, and dC are one standard deviation (68% confidence interval) of N_{e0}, D, and C based on the least-square fitting with the O mode propagation model.

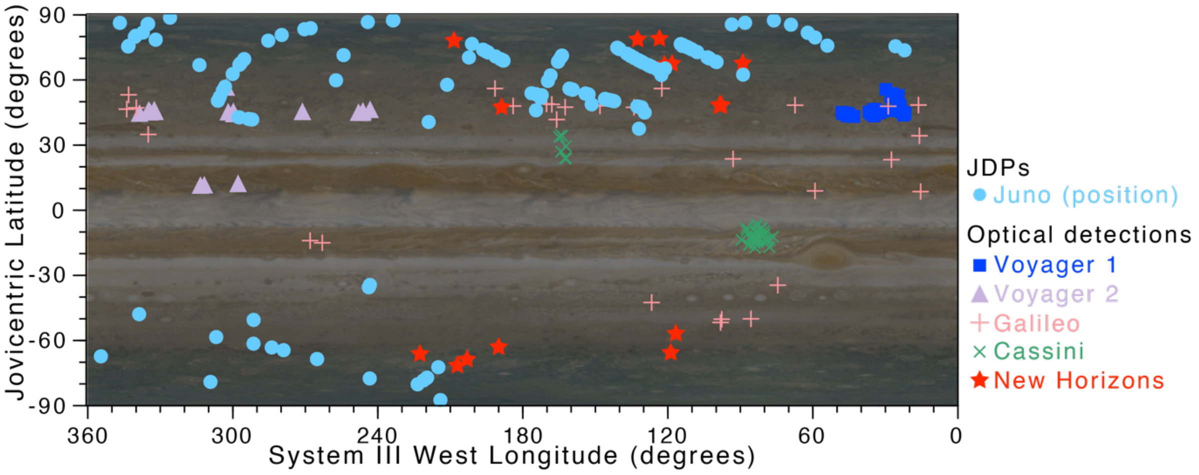

Supplementary Figure 10 | Global distribution map of Jupiter's lightning. The locations of 445 detections of JDPs are compared with the optical detections made by Voyager 1 (36 lightning locations)[1], Voyager 2 (18 lightning locations)[2], Galileo (estimated 336 flashes in 28 storms)[3,4], Cassini (50 flashes in four spots)[5], and New Horizons (18 flashes)[6]. The corresponding symbols and colours indicate in the legends on the right side. The Jovian image was provided by NASA/JPL-Caltech/SSI/SwRI/MSSS/ASI/INAF/JIRAM/Björn Jónsson (http://www.planetary.org/multimedia/space-images/jupiter/merged-cassini-and-juno.html).

References

1. Williams, M. A. An analysis of the Voyager images of jovian lightning *The University of Arizona* (1986).
2. Borucki, W. J. & Magalhães, J. A. Analysis of Voyager 2 images of Jovian lightning *Icarus* **96**, 1-14 (1992).
3. Little, B. *et al.* Galileo Images of Lightning on Jupiter *Icarus* **142**, 306-323 (1999).
4. Gierasch, P. J. *et al.* Observation of moist convection in Jupiter's atmosphere *Nature* **403**, 628-630 (2000).
5. Dyudina, U. A. *et al.* Lightning on Jupiter observed in the H_α line by the Cassini imaging science subsystem *Icarus* **172**, 24-36 (2004).
6. Baines, K. H. *et al.* Polar Lightning and Decadal-Scale Cloud Variability on Jupiter *Science* **318**, 226-229 (2007).
